# Rapid and High-Fidelity Fabrication of Embedded Elastomeric Photomask for Wafer-Scale Sub-Micrometer Conformal Contact Photolithography

**DOI:** 10.3390/mi17040456

**Published:** 2026-04-08

**Authors:** Huikang Liang, Bingquan Lei, Zhiwen Shu, Lei Chen, Huigao Duan

**Affiliations:** 1 College of Mechanical and Vehicle Engineering, National Engineering Research Centre for High Efficiency Grinding, Hunan University, Changsha 410082, China; huikang_liang@hnu.edu.cn (H.L.); bingquanlei@hnu.edu.cn (B.L.); shuzhiwen@hnu.edu.cn (Z.S.); 2 Engineering Product Development, Singapore University of Technology and Design, Singapore 487372, Singapore; lei_chen@sutd.edu.cn

**Keywords:** contact photolithography, embedded elastomeric mask, wafer-scale sub-micrometer fabrication, cost-effective fabrication, rapid process

## Abstract

Photolithography is the mainstream technology used in micro/nanofabrication. While projection photolithography is widely used in production, with a resolution close to the wavelength of the light source, its processes are complicated and expensive. Moreover, in projection photolithography, scanning and splicing are required to achieve large-area exposure at the wafer level, which reduces throughput in production. Contact photolithography offers a cost-effective and parallel exposure solution, but achieving uniform resolution over large areas with micrometer or sub-micrometer resolution remains a challenge. In this study, we propose a conformal contact photolithography strategy based on a wafer-scale embedded elastomeric mask. By optimizing metal patterning and embedding transfer processes, we significantly improve the area (wafer-scale) and efficiency (lift-off and metal transfer process within seconds) of metal-embedded elastomeric mask fabrication. This method enables the rapid and cost-effective fabrication of large-area sub-micrometer-resolution structures, with broad applications in the production of sub-micrometer devices and academic research.

## 1. Introduction

Photolithography is the cornerstone of semiconductor manufacturing and a fundamental technology for the fabrication of micro/nanodevices [1]. Presently, projection photolithography dominates production, scaling mask patterns to achieve exposure with resolutions close to or exceeding the wavelength of the light source [2,3,4,5]. However, to use this method, a complex and expensive photolithography system is required. Furthermore, projection photolithography is limited by its single-exposure area, necessitating the use of a step-by-step scanning method to achieve wafer-level patterning [6,7], which reduces throughput in production. Therefore, a more cost-effective and efficient photolithography method will have significant application potential.

**Figure A1:**
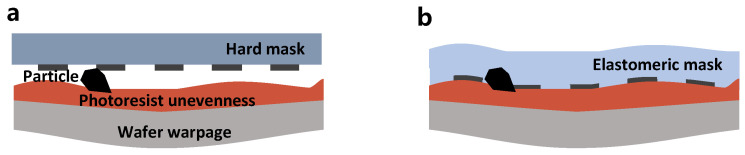
Schematic images of the contact interface between mask and photoresist: (**a**) hard mask; (**b**) elastomeric mask.

Contact photolithography, an early photolithography method used in semiconductor manufacturing, provides a cost-effective and parallel exposure solution through 1:1 pattern transfer by directly contacting the mask with the photoresist [8]. Its primary limitation is resolution, as conventional contact photolithography achieves single-micrometer-level resolution [9,10]. During wafer-level photolithography, due to issues such as wafer warping, flatness, photoresist unevenness, and particle contamination, among others, it is difficult to ensure close contact between the hard mask and photoresist everywhere (as shown in Figure A1). The gap between the mask and the photomask surface will cause diffraction effects, resulting in deviations in the light intensity distribution from the ideal situation [11] and degrading resolution [12]. The influence of the gap on the resolution is given by the following equation [13,14]:

$$CD=\frac{3}{2}\sqrt{\lambda (g+\frac{t}{2})}$$

where *CD* is the critical dimension, *λ* is the exposure wavelength, *g* is the gap between the mask and photoresist, and *t* is the thickness of photoresist.

To solve the contact issue, researchers have developed various photolithography masks. In terms of structure, the embedded design can make the mask fit the photoresist more closely while reducing mechanical damage during contact. This design can also increase the lateral constraint of the mask on light intensity, thereby improving photolithography resolution. The ultra-thin embedded hard mask, combined with vacuum assistance, can achieve compatibility with substrate warpage [15,16,17], but it is susceptible to the flatness and particle contaminants of the substrate surface. In terms of the mask substrate material, the elastomeric masks can establish effective contact with photoresist due to their adaptability, enabling contact photolithography with a near-zero gap. The masks made of purely elastomeric materials are limited by the optical confinement capability and the design freedom of the patterns [18,19,20]. The embedded elastomeric masks combine the advantages of an embedded design and can achieve perfect contact with the surface of the photoresist without any restrictions in terms of pattern design. It is an excellent contact photolithography mask solution, and carbon black [21,22,23] and metals [10,24,25] are commonly used embedding materials. Among them, the metal-based elastomeric mask exhibits better mechanical and chemical stability. However, the fabrication of metal-based elastomeric masks [10] remains time-consuming (metal transfer ~several days) and area-limited (~1 mm × mm). It is necessary to improve the fabrication process of the masks to make it possible to achieve wafer-scale micrometer/sub-micrometer contact photolithography.

In addition to the reported fabrication methods of metal-embedded elastomeric masks, some works of patterning metals on flexible substrates [26,27,28,29,30] also seem to have potential in mask fabrication, although their applications are mainly focused on flexible electronic devices. However, in reality, these methods are unable to be used for micrometer/sub-micrometer masks due to the limitations of pattern resolution [26], edge roughness [27,28,29], and the incompatibility of embedded design [30].

In this study, we propose a reliable, large-area conformal contact lithography scheme based on embedded-metal elastomeric masks. Using peelable bottom-cut photoresist, we achieve reliable patternization of low-adhesion metal (~100%). With PI-PDMS composite materials used as elastomeric substrates, we achieve rapid mechanical stripping for embedding and transferring patterned metals (within seconds), enabling the rapid fabrication of large-area (wafer-scale) embedded-metal elastomeric masks. Compared with the reported embedded-metal elastomeric mask fabrication methods, our scheme demonstrates advantages in terms of mask area and fabrication efficiency, which means lower cost and higher efficiency for exposure. This rapid and large-area contact photolithography scheme has significant application potential for the batch production of sub-micrometer-resolution devices.

## 2. Materials and Methods

### 2.1. Fabrication of Embedded-Metal Elastomeric Masks

A ~500 nm layer of modified LOR 3A photoresist (Kayaku Advanced Materials, Inc., Westborough, MA, USA) and a ~500 nm layer of AZ1500 photoresist (Kayaku Advanced Materials, Inc., Westborough, MA, USA) were spin-coated and prebaked on a SiO_2_ substrate. The modified LOR 3A configuration involved adding 0.002 g of PMAFP to 10 g of LOR 3A. After stirring for 0.5 h, the modified LOR 3A was stored in a dark environment for 24 h. A photoresist pattern with undercutting was obtained using laser direct writing lithography (MiScan200, SVG Tech Group Co., Ltd., Suzhou, China), and it was developed using an RZX-3038 developer (Ruihong (Suzhou) Electronic Chemicals Co., Ltd., Suzhou, China) for 25 s. After photoresist patterning, 10 nm of Ag was deposited first via vacuum thermal evaporation (JSD300, Anhui Jiashuo Vacuum Technology Co., Ltd., Hefei, China), followed by 40 nm of Cr. A vacuum film-sticking platform was used to apply the tape to the surface of the evaporated sample, which was then stripped off to obtain clean metal patterns.

Next, a PI resin with imidination treatment was spin-coated and baked at 85 °C for 0.5 h. After curing, an ~800 nm thick PI layer was obtained. Then, UV-curing adhesive was spin-coated, and PDMS resin (Sylgard184, Dow Corning, Midland, MI, USA) was poured onto the sample with metal patterns. The PDMS resin was prepared via mixing PDMS and a cross-linker (10:1 *w*/*w*) and degassing under vacuum. After waiting for 5 min for the underlying PDMS to initially cure, the sample was exposed under 365 nm ultraviolet light for 10 s to ensure full bonding between the PI and the initially cured PDMS. Following UV treatment, the sample was baked at 80 °C for 2 h to fully cure the PDMS. A blade was used to cut along the edge of the substrate to assist with separating the elastomeric mask from the substrate. Finally, the entire film was completely stripped from the substrate to obtain an elastomeric mask.

### 2.2. Contact Photolithography Using Elastomeric Masks

AZ1500 photoresist was spin-coated and prebaked on a SiO_2_ substrate. An elastomeric mask was then stuck on the sample. Before sticking, the elastomeric mask was treated with ozone for 5 min to enhance its surface energy and reduce bubble formation during the sticking process. The sample with the elastomeric mask was exposed using a 365 nm ultraviolet lithography platform. After development, the patterns on the elastomeric mask were replicated onto the new substrate.

### 2.3. Fabrication of Transparent Metal Film

A peelable modified polyvinyl alcohol (PVA) photoresist (negative) was spin-coated on a SiO_2_ substrate. After sticking the elastomeric mask, the PVA photoresist was exposed using a 365 nm ultraviolet lithography platform and developed in water to obtain PVA patterns. Subsequently, 100 nm of Ag was deposited via vacuum thermal evaporation. The Ag mesh structures were obtained by dry lift-off using tape. The PI solution was spin-coated on the Ag mesh, baked to form a film, and then stripped from the substrate to obtain a transparent metal film, which can be used as a heating device.

### 2.4. Characterization

Morphology characterization was conducted using an optical microscope (Axiolab 5, Carl-ZEISS, Oberkochen, Germany), confocal laser scanning microscope (LEXT OLS5100, Olympus Corporation, Hachioji-shi, Japan), and field-emission scanning electron microscopy (SEM) (SIGMA-HD, Carl-ZEISS, Oberkochen, Germany). The optical transmittance was characterized using a spectrophotometer (UV-2600i, Shimadzu Corporation, Nakagyo-ku, Japan).

## 3. Results and Discussion

**Figure 1:**
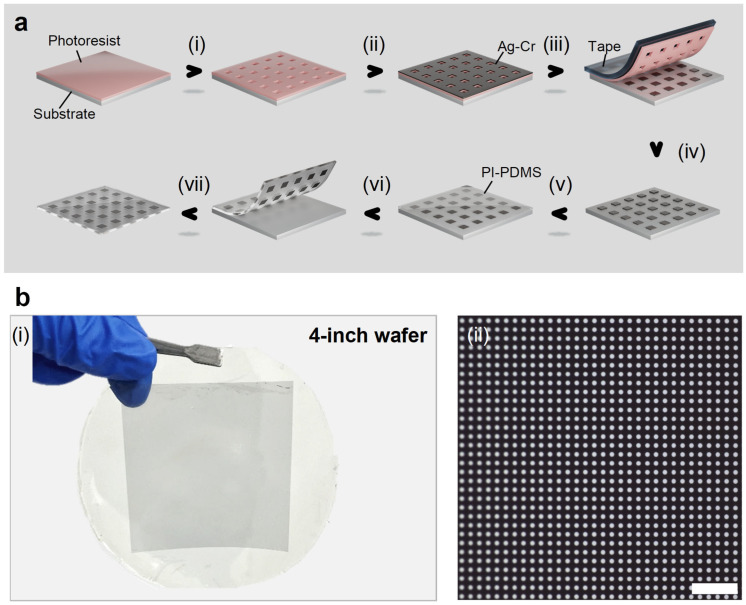
Rapid fabrication flow of elastomeric masks and exhibition of a large-area result. (**a**) Schematic images of the process flow. (**i**) Laser direct writing lithography; (**ii**) metal deposition; (**iii**) stripping; (**iv**) finishing the metal patterning; (**v**) spin-coating and curing of the PI-PDMS composite material; (**vi**) stripping; (**vii**) obtaining the embedded-metal elastomeric mask. (**b**) Four-inch elastomeric mask fabricated using our process. (**b**(**i**)) Optical image of the elastomeric mask. (**b**(**ii**)) Detailed image of the structures in the elastomeric mask. (**b**(**i**)). Scale bars, 20 μm in (**b**(**ii**)).

To achieve near-zero gap contact lithography and ensure uniformity over large areas, we developed a rapid fabrication method for embedded-metal elastomeric masks, as shown in Figure 1a. Firstly, double-layer peelable photoresist was spin-coated on the substrate and patterned using laser direct writing lithography (step i). A weak-adhesion Ag layer and light-absorbing Cr layer were then deposited (step ii). Due to the undercutting of the photoresist and weak-adhesion treatment, the photoresist and excess metal can be completely stripped off (step iii) using tape, leaving the weak-adhesion metal structures intact (step iv). Then, PI and PDMS were solidified (step v) on the substrate surface to ensure that the metal structures were completely embedded in the PI-PDMS composite elastomeric material. Finally, the elastomeric layer was stripped from the substrate (step vi) to form the embedded-metal elastomeric mask (step vii). Figure 1b(i) shows a 4-inch embedded elastomeric mask fabricated using our process. The mask pattern consists of a disk array with a diameter of 2 μm and a pitch of 4 μm. As seen in Figure 1b(ii), the structures in the elastomeric mask are intact, with no missing parts or pattern distortion. Unlike the multi-day (~1 mm × 1 mm) Ni sacrificial layer method reported by Sanyoon Paik et al. [10], using our process, embedded-metal elastomeric masks are achieved within seconds via mechanical peeling after PDMS curing. Furthermore, the efficiency of the sacrificial layer method is related to the mask area; this means that the fabrication of larger masks may take a week or even longer, which makes it almost impossible to achieve wafer-scale elastomeric masks using this method. However, the mask area has no significant impact on our metal transfer process. Only a few seconds of mechanical stripping are required for the 4-inch metal transfer.

The core challenge in the fabrication of embedded-metal elastomeric masks is whether elastomeric masks and metal patternization processes are compatible. Therefore, achieving metal patternization on a hard substrate and transferring the metal structures to an elastomeric substrate comprises a suitable fabrication process. However, chromium (Cr), a commonly used light-absorbing layer material, often exhibits strong adhesion to the substrate, making it difficult to ensure the transfer of intact Cr structures. To overcome this, with our process, we incorporate a low-adhesion Ag layer between the substrate and Cr layer, ensuring structural integrity during the transfer process. However, traditional wet lift-off results in low yields for low-adhesion metal structures, as these structures are prone to loss during the wet process. Additionally, residue issues cannot be alleviated (such as ultrasonic) due to the weak adhesion characteristic, making wet lift-off unsuitable for Ag-Cr patterning in our process.

**Figure A2:**
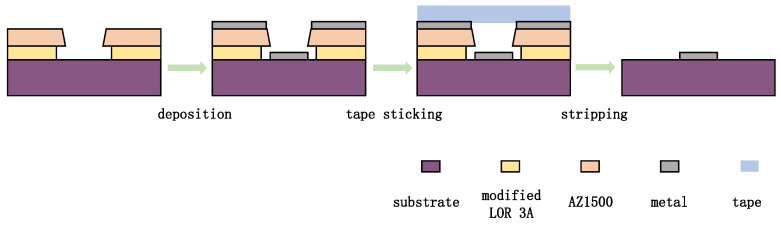
Process flow of double-layer photoresist dry lift-off.

To address these challenges, we developed a double-layer dry lift-off process based on peelable photoresist. This solution stems from two considerations: firstly, it utilizes the peelable property to achieve dry lift-off, reducing structure loss and residue issues while improving yield compared with wet lift-off. Secondly, during the photolithography process, the exposure of a single layer of positive photoresist is affected by the diffraction effect, leading to a top-cutting trend in the cross-sectional morphology of the photoresist. This can result in continuous films forming on the sidewalls during metal deposition, reducing lift-off yield. To mitigate this, in our process, a double-layer photoresist combination of LOR 3A (with low-adhesion treatment) and AZ1500 is employed to form sufficient undercutting, ensuring the independence of the photoresist surface material from the metal structures on the substrate. Figure A2 illustrates the cross-sectional changes in the double-layer photoresist during the dry lift-off process.

**Figure A3:**
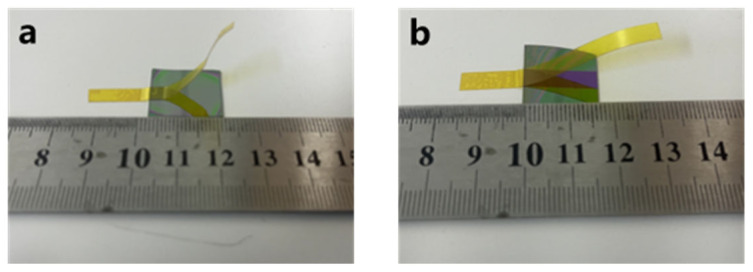
Comparison of stripping performance of LOR 3A photoresist with and without modification. Stripping results of unmodified (**a**) and modified (**b**) LOR 3A photoresist on SiO_2_ substrate.

Similarly to our previous works [31], we incorporated polyether-modified acrylic functional polydimethylsiloxane (PMAFP) amphiphilic molecules into the LOR 3A to obtain a modified photoresist with weak adhesive properties. As shown in Figure A3, the stripping performance of the modified photoresist significantly differs from that of the original LOR 3A, transitioning from non-peelable (LOR 3A) to peelable (modified photoresist). To quantify and evaluate the stripping performance of the modified photoresist, we analyzed the energy release rate *G* based on linear elastic fracture mechanics. The energy release rate represents the conditions for crack propagation, with a larger *G* value indicating greater difficulty in crack propagation. The energy release rate is expressed as follows [32,33]:

$$G=\frac{F}{w}\left(1-\cos\phi \right)$$

**Figure 2:**
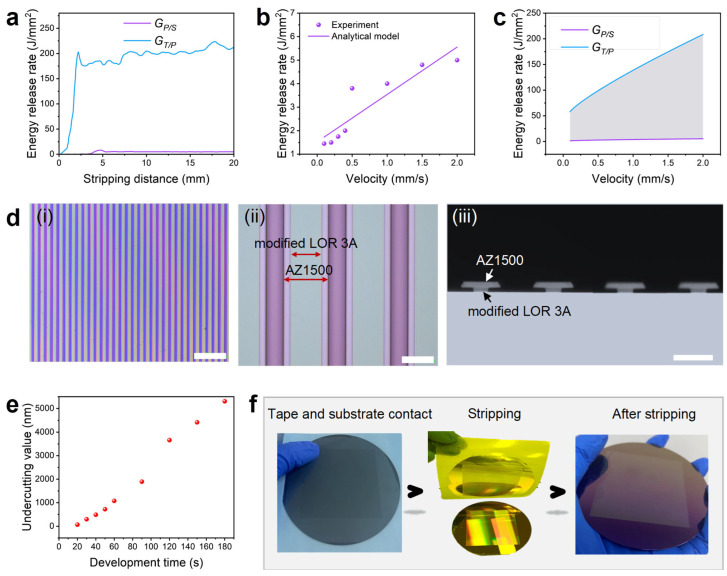
Study of process parameters of double-layer photoresist dry lift-off and exhibition of large-area results. (**a**) Test results of energy release rates at the interfaces of the modified photoresist/SiO_2_ substrate and photoresist/tape, with stripping velocity of 2 mm/s. (**b**,**c**) Energy release rates of the two interfaces at different stripping velocities. Among them, (**b**) is the energy release rate of photoresist/SiO_2_ substrate with a small *Y*-axis coordinate system. (**d**) Grating structures fabricated by double-layer photoresist dry lift-off. The patterns are a 1:1 grating with a line width of 20 μm. (**d**(**i**,**ii**)) Optical images of the grating structures. (**d**(**iii**)) SEM cross-section image of grating structures. (**e**) Relationship between development time and undercutting value. (**f**) Optical images of each step of the 4-inch wafer dry lift-off. Scale bars, 200 μm in (**d**(**i**)), 20 μm in (**d**(**ii**)), and 10 μm in (**d**(**iii**)).

In this formula, *F* represents the peeling force, *w* represents the width of the interface, and *ϕ* represents the peeling angle. During the dry lift-off process, the adhesive tape is applied to the photoresist surface, creating two interfaces: tape/photoresist (T/P) and photoresist/substrate (P/S). When

$$G_{T/P}>G_{P/S},$$

the photoresist can be stripped from the substrate surface. Through stripping tests, we obtained the *G* value of the two interfaces, as shown in Figure 2a. The *G_P/S_* corresponding to the modified photoresist is significantly smaller than *G_T/P_*, enabling easy stripping of the modified photoresist from the substrate. The substrate surface after stripping is clean, with no residual photoresist. To ensure the stability of the stripping process, we also tested the energy release rates of the two interfaces at different stripping velocities, as shown in Figure 2b,c. The results demonstrate that even at very low stripping velocities, there is at least an order of magnitude difference between *G_P/S_* and *G_T/P_*, confirming the stable stripping performance of the modified photoresist.

**Figure A4:**
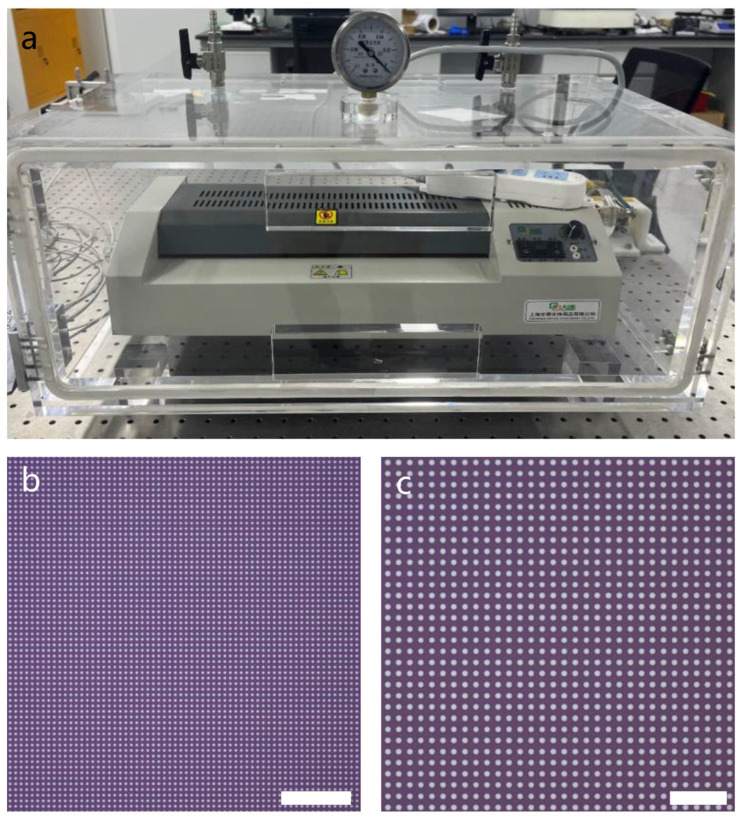
Self-built vacuum film sticking platform and result of the 4-inch wafer dry lift-off. (**a**) Optical image of the vacuum film-sticking platform. (**b**,**c**) Microscopic images after dry lift-off; the designed pattern is an array of disks with a 2 μm diameter and a 4 μm pitch. Scale bars, 50 μm in (**b**) and 20 μm in (**c**).

A peelable undercut photoresist structure is achieved using a double-layer photoresist configuration, with AZ1500 as the upper layer and the modified photoresist as the lower layer. Due to the properties of LOR 3A, which does not require exposure and dissolves faster in the developer, a undercut structure can be formed in a single development step. These properties are retained in the modified photoresist. Figure 2d(i–iii) shows the grating morphology using the double-layer photoresist. The semi-transparent edge formed by the undercutting photoresist is visible in the optical image (Figure 2d(ii)), while the undercutting morphology is more clearly observed in the SEM cross-section image (Figure 2d(iii)), where the undercutting value reaches several micrometers. However, for higher-resolution patterns, precise control of the undercutting value is necessary to prevent pattern collapse, slippage, or sidewall contamination. As shown in Figure 2e, we studied the relationship between development time and undercutting value, ultimately selecting a development time of 25 ss for subsequent metal patterning processes, resulting in an undercutting depth of ~70 nm. This dry lift-off solution demonstrates exceptional performance in large-area micrometer- and sub-micrometer-resolution metal patternization. Figure 2f shows the optical images of each step of the 4-inch wafer dry lift-off process using a self-built vacuum film sticking platform (Figure A4). The entire film-sticking–stripping process was completed in just a few minutes, yielding weakly adherent metal structures that are intact without any missing parts, excess photoresist, or metal residue, as shown in Figure A4. Compared with the wet lift-off process, which takes several hours to a full day to complete, it offers a significant improvement in efficiency.

**Figure A5:**
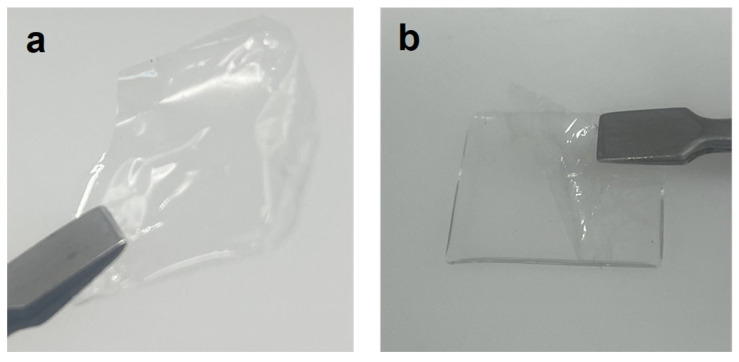
Problems exist in PI films. (**a**) Single-layer PI curls due to stress. (**b**) Delamination between PI and PDMS.

**Figure A6:**
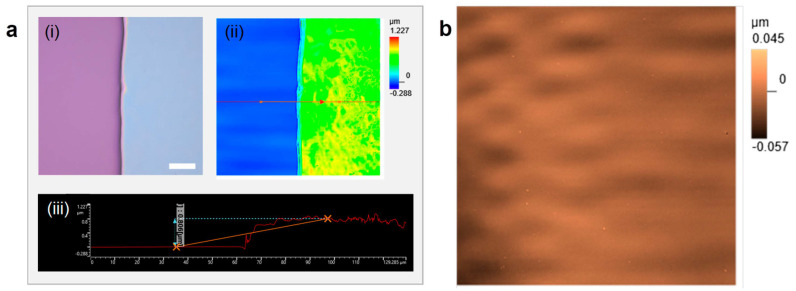
Characterization of PI layer. (**a**) Characterization of PI film thickness. (**a**(**i**)) Optical image of the PI-SiO_2_ substrate step. (**a**(**ii**,**iii**)) Thickness scanning via laser confocal microscopy. (**b**) Surface morphology of PI scanned using laser confocal microscopy. Scaled bar, 20 μm in (**a**(**i**)).

**Figure A7:**
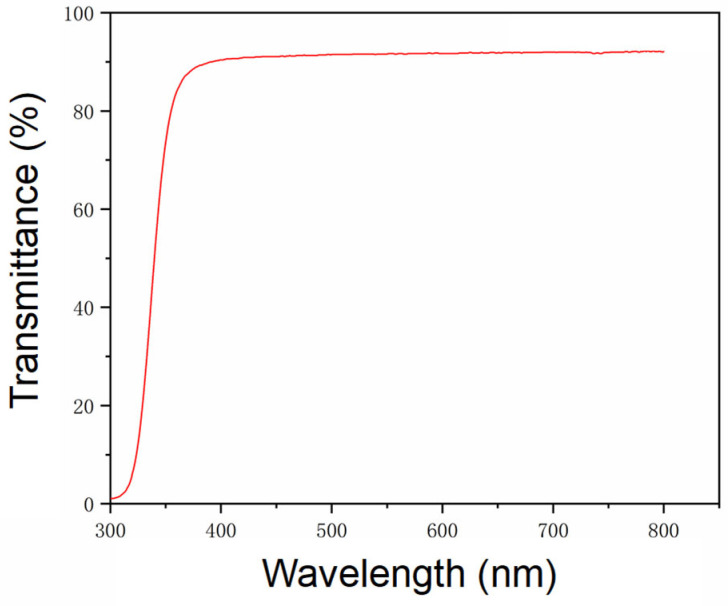
Transmittance of the PI-PDMS composite material at wavelengths of 300–800 nm.

After the patterning of weakly adherent metal structures, the next challenge is embedding and transferring these structures onto an elastomeric substrate to fabricate the mask. A significant issue arises from the low Young’s modulus of PDMS, which results in weak adhesion between traditional PDMS and metals. Even for metals with weak adhesion to the substrate, achieving successful stripping is still challenging. To address this, we selected PI, a material with greater plasticity, as the embedding layer to ensure intact transfer of the metal structures. PDMS serves as the carrier film, providing elastic capacity. However, a single PI layer may curl due to large curing stress, as shown in Figure A5, which is another reason for using composite elastomeric substrates. The spin-coated PI is extremely thin (only 800 nm (Figure A6)) compared with PDMS, minimizing its impact on the elastic performance of the elastomeric mask. Additionally, since the PI film was cured in situ on a SiO_2_ substrate with extremely low roughness, the resulting elastomeric mask has a smooth surface with nanometer-level average roughness (Figure A6), ensuring near-zero contact between the elastomeric mask and the photoresist. It should be noted that the adhesion between PI and PDMS is relatively weak, as shown in Figure A5. To prevent delamination, we used UV-curing adhesive for bonding. After determining this combination, we measured the light transmittance of the final elastomeric composite material (Figure A7) to ensure that the fabricated elastomeric mask has good photolithography efficiency and exposure resolution. The light transmittance of the elastomeric composite material reaches 87.6% at a wavelength of 365 nm, making it suitable for contact photolithography.

**Figure 3:**
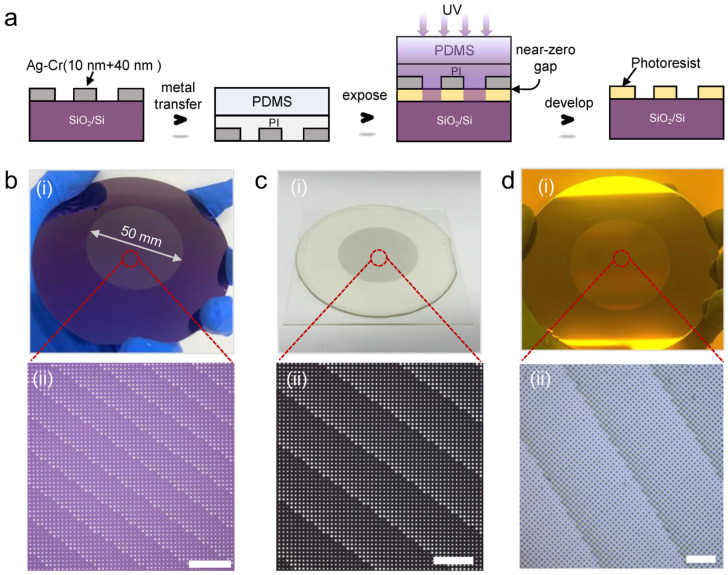
Fabrication and exposure of an elastomeric mask for 50 mm aperture metalenses. (**a**) Fabrication and photolithography flow of the elastomeric mask. (**b**) Optical images of metal structures after dry lift-off. (**c**) Elastomeric mask. (**d**) Conformal contact photolithography results. (**b**(**i**),**c**(**i**),**d**(**i**)) Actual images. (**b**(**ii**),**c**(**ii**),**d**(**ii**)) Optical microscope images. Scale bars, 50 μm in (**b**(**ii**),**c**(**ii**)) and 20 μm in (**d**(**ii**)).

**Figure A8:**
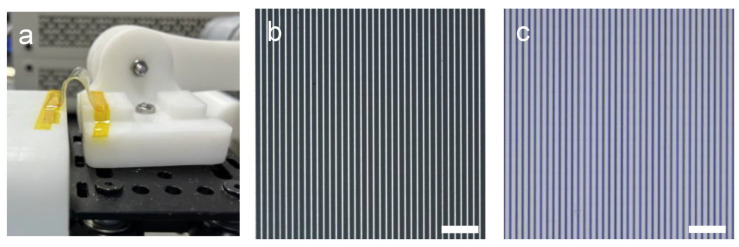
Mechanical stability test of an elastomeric mask. (**a**) Bending test platform. (**b**) Optical image of elastomeric mask after 5000 bends. (**c**) Optical image of the lithography result after 5000 bends. Scaled bars, 20 μm in (**b**,**c**).

Using the abovementioned dry lift-off process and elastomeric composite material, we can achieve rapid fabrication of embedded-metal elastomeric masks for large-area contact photolithography. Figure 3a illustrates the cross-sectional changes that occurred throughout the mask fabrication and photolithography process. As shown in Figure 3b(i,ii), we fabricated a 50 mm aperture metalens metal structure using the dry lift-off process. The metalens consists of disk patterns with diameters ranging from 1.2 μm to 2.5 μm. The obtained metal structures are intact across the entire wafer, with no loss or excess residue. The elastomeric mask of the metalens was then fabricated by transferring the metal structures in Figure 3b(i,ii) using the composite elastomeric material. As shown in Figure 3c(i,ii), the structures remain intact, with no loss or distortion after the transfer process. Finally, conformal contact photolithography was performed using the fabricated elastomeric mask with 365 nm of UV light. The photolithography result shown in Figure 3d indicates that the fabricated wafer-scale elastomeric mask has excellent exposure capability, and the obtained 1–2 μm resolution structures with clear edges have reached the limit resolution of traditional hard masks. To evaluate the durability of the mask, we conducted a bending test, as shown in Figure A8. The result indicates that the metal patterns in the embedded elastomeric mask have no obvious fracture or loss after 5000 bends, and the exposed result demonstrates a very good ability to maintain the exposure resolution before and after the bending test.

**Figure 4:**
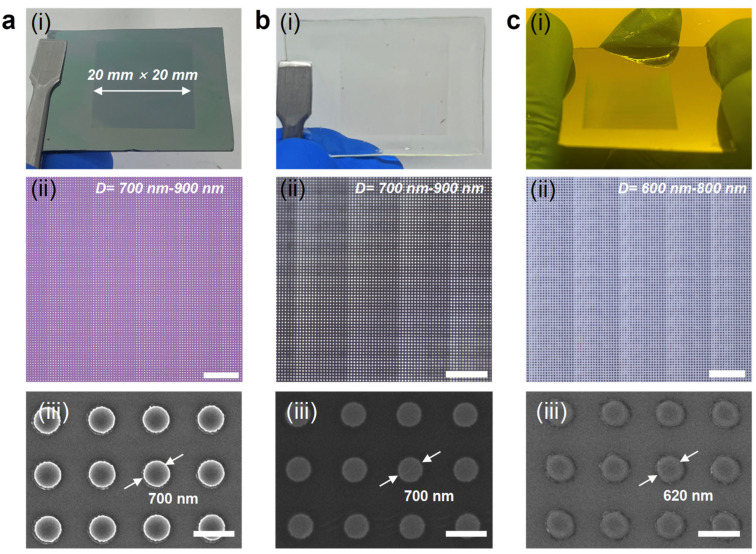
Fabrication and exposure of sub-micron-resolution elastomeric mask. (**a**) Dry lift-off results. (**b**) Elastomeric mask. (**c**) Conformal contact photolithography results. (**a**(**i**),**b**(**i**),**c**(**i**)) Physical images. (**a**(**ii**),**b**(**ii**),**c**(**ii**)) Optical microscope images. (**a**(**iii**),**b**(**iii**),**c**(**iii**)) SEM images. Scale bars, 20 μm in (**a**(**ii**)–**c**(**ii**)) and 1 μm in (**a**(**iii**)–**c**(**iii**)).

**Figure A9:**
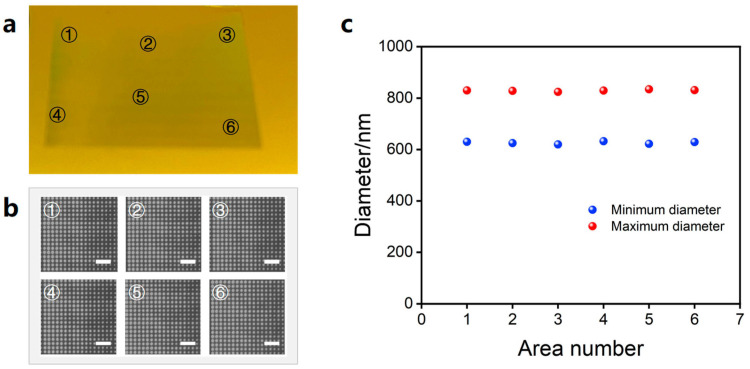
Uniformity test of sub-micrometer structure exposure. (**a**) The positions of the selected 6 test points. (**b**) SEM images of the 6 positions. (**c**) Statistical results of the maximum and minimum diameters at the 6 positions. Scale bars, 5 μm in (**b**).

**Figure A10:**
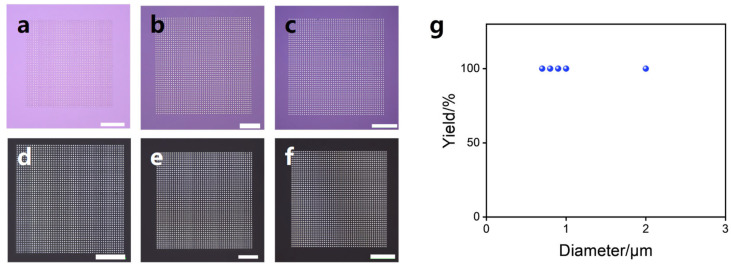
Yield test of the mask fabrication process. Dry lift-off results of 2500 different-sized disk arrays, with diameters ranging from 700 to 900 nm (**a**), 1 μm (**b**), and 2 μm (**c**). Metal embedding transfer results, with diameters ranging from 700 to 900 nm (**d**), 1 μm (**e**), and 2 μm (**f**). (**g**) The overall yield statistical result of the two steps. Scale bars, 20 μm in (**a**,**b**,**d**,**e**) and 50 μm in (**c**,**f**).

Our method is also capable of rapidly fabricating sub-micrometer-resolution structures, achieving resolution close to the limit of conventional contact photolithography using a hard mask. We designed a sub-micrometer pattern array to verify this capability. Figure 4a(i)–c(i) displays the fabricated metal structures, the elastomeric mask, and the photoresist patterns obtained using contact photolithography, respectively. The corresponding optical and SEM images with detailed morphology are shown in Figure 4a(ii)–c(ii),a(iii)–c(iii). The designed pattern consists of a disk array with diameters ranging from 700 to 900 nm, a pitch of 1.4 μm, and an array area of 20 mm × 20 mm (~1in wafer). After contact photolithography, the photoresist pattern critical dimension (CD) is slightly smaller than that on the mask, at approximately 600–800 nm. This discrepancy is attributed to the effects of reflected light during exposure and limited photoresist development contrast. With the measured maximum and minimum CD at six different positions (Figure A9) displayed in Figure 4c(i), we confirmed that an embedding-metal elastomeric mask can achieve excellent uniformity in contact photolithography. Moreover, in order to quantify the yield of our mask fabrication process, we tested the yield of dry lift-off and metal transfer at different resolutions, as shown in Figure A10. The results show that the yield of the two steps reached 100% within the resolution range of 700 nm to 2 μm. These results demonstrate that when using our process, high-yield and rapid fabrication of large-area sub-micrometer elastomeric masks can be achieved, with excellent exposure uniformity. Although the reported limit of photolithography resolution of elastomeric masks is several tens of nanometers [10], the realization of this high resolution relies on the control of the photoresist development process, making the achievement of this high resolution while freely controlling the morphology of the pattern difficult. In contrast, within sub-micrometer resolution, the use of conformal contact photolithography based on elastomeric masks can result in the achievement of a replication closer to 1:1 with the mask pattern, offering higher pattern precision control capabilities and meeting the application requirements of manufacturing devices.

**Figure 5:**
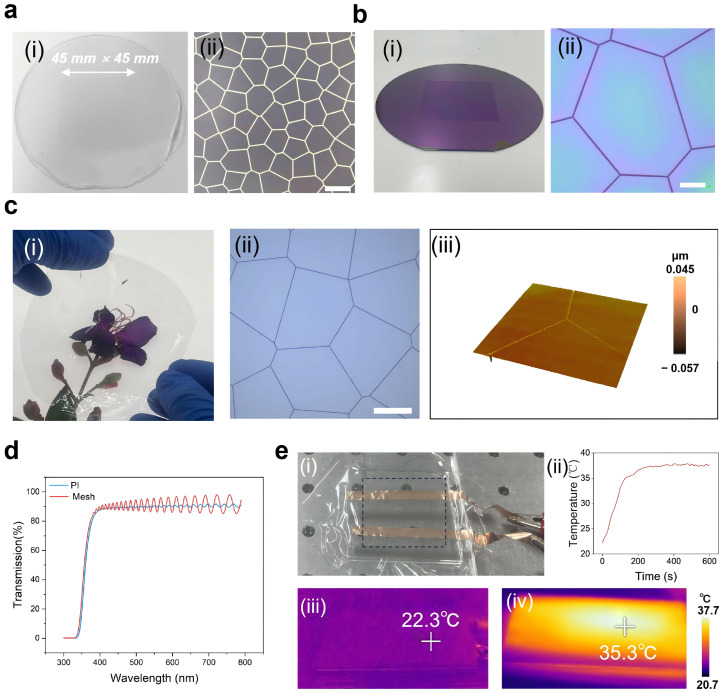
Transparent wafer-scale mesh fabricated by our method. (**a**) Random mesh elastomeric mask. (**b**) Exposure result of the elastomeric mask. (**c**) Transparent metal film. (**a**(**i**),**b**(**i**),**c**(**i**)) Physical images. (**a**(**ii**),**b**(**ii**),**c**(**ii**)) Optical images. (**c**(**iii**)) Three-dimensional scanning result of the transparent metal mesh surface. (**d**) Result of light transmittance test. (**e**) Heating performance of the transparent metal film. (**e**(**i**)) Physical image of the heating test platform. The dashed box represents the mesh area. (**e**(**ii**)) Heating rate of transparent metal film. (**e**(**iii**)) Temperature distribution of the sample before heating. (**e**(**iv**)) Temperature distribution of the sample after heating for 2 min. Scale bars, 100 μm in (**a**(**ii**)), 20 μm in (**b**(**ii**)), and 50 μm in (**c**(**ii**)).

Using our conformal contact photolithography scheme, we can achieve rapid small-batch fabrication of large-area sub-micrometer devices. For example, we fabricated transparent metal films using elastomeric masks. As shown in Figure 5a(i), we fabricated a random mesh elastomeric mask with a 4-inch wafer. Figure 5a(ii) presents the detailed morphology of the elastomeric mask, with a line width of 1.2 μm and a pitch of approximately 120 μm. Using the elastomeric mask shown in Figure 5a(i), a peelable PVA negative photoresist [32] was exposed with 365 nm UV light to obtain the random mesh photoresist pattern shown in Figure 5b(i,ii), with a measured line width of 1.1 μm. Subsequently, metal random mesh structures were fabricated on a hard substrate using deposition and dry lift-off processes. Due to the stable performance of PI under high-temperature and high-humidity conditions, we selected it as the base material for the transparent heating film of metal mesh. The mesh transfer process is similar to the elastomeric mask transfer process, and ultimately, a PI film embedded with metal mesh was obtained; the obtained transparent metal film is shown in Figure 5c(i). Using the transparent metal film, the flowers behind the film can be clearly observed, and the metal mesh is difficult to observe with the naked eye. Moreover, from the optical microscope and three-dimensional morphology scan images of Figure 5c(ii,iii), it can be seen that the metal structure on the PI film is intact, with no fractures or missing parts. The metal structures are flush with the PI surface and well embedded in the PI film. To quantify the light transmission performance of the transparent mesh, we measured the film transmittance in the visible light band with and without the metal mesh, as shown in Figure 5d. The transmittance with the metal mesh exceeds 80%, with only a slight decrease compared with the transmittance without metal mesh (~90%), meeting the application requirements for devices with transparency demands.

The fabricated transparent metal mesh film can be used as a heating element in applications requiring transparency and thinness, such as glass defogging, where the temperature must be stabilized above the dew point. We attached the transparent metal film to a small test platform (Figure 5e(i)) and tested the heating performance of the film using a thermal infrared camera. The temperature changes at the center of the mesh within 10 min after applying a 4V voltage were recorded using the camera, as shown in Figure 5e(ii). Figure 5e(iii,iv) shows the temperature distribution at the beginning and after 2 min of heating. The results demonstrate that the transparent metal film fabricated using our method achieves a rapid temperature increase (~2 min) from room temperature (22 °C) to 35 °C, and this can be stably maintained at 37 °C, fulfilling the requirements for efficient defogging functionality.

## 4. Conclusions

Using our process to achieve the rapid fabrication of an embedded elastomeric mask, we have demonstrated that our conformal contact photolithography technology is a highly cost-effective solution for micrometer/sub-micrometer wafer-scale photolithography. The reliable dry lift-off process and mechanically peelable embedded-metal structures enable extremely high fabrication efficiency, making large-area elastomeric mask fabrication feasible. Although we only demonstrated a sample of ~1 in the wafer at sub-micrometer resolution, this was due to the limitations of our laser direct writing equipment. If we replace the direct writing equipment with that which displays a higher performance, using our process, the fabrication of elastomeric masks with sub-micrometer resolution and a larger area is achievable. Our technology has broad application prospects in the fabrication of devices with large-area micro/nanostructures, especially in the fields of micro/nanostructure functional films, metalenses, and other devices.

## Data Availability

The original contributions presented in this study are included in the article. Further inquiries can be directed to the corresponding author.
